# Knowing one’s place? The role of income inequality in shaping positioning bias across 29 countries

**DOI:** 10.3389/fsoc.2025.1617014

**Published:** 2026-01-06

**Authors:** Timo Wiesner, Julio Iturra-Sanhueza

**Affiliations:** 1 SOCIUM — Research Center on Inequality and Social Policy, University of Bremen, Bremen, Germany; 2 Bremen International Graduate School of Social Sciences (BIGSSS), University of Bremen, Bremen, Germany

**Keywords:** inequality perceptions, income inequality, subjective social status, segregation, multilevel modelling

## Abstract

This study investigates how individuals’ misperceptions of their relative position in the income distribution—referred to as positioning bias—are shaped by income inequality. Drawing on the ISSP 2019 Social Inequality module, the analysis includes data from 31,368 individuals across 29 countries and employs multilevel modelling to test whether individuals are more likely to misperceive their position under conditions of high inequality. We explore heterogeneity across income groups and bias types (unbiased, underestimation, and overestimation). Findings reveal that inequality is associated with positioning bias, though the direction and magnitude depend on the individuals’ actual income position. Individuals in disadvantaged positions are more accurate in their self-perceptions when inequality is high, while those in higher-income positions tend more towards underestimating their relative standing. Overall, the results show that higher inequality is associated with lower subjective status relative to respondents’ actual position across all income groups. This research contributes to broader debates on inequality perceptions and redistributive preferences.

## 1 Introduction

One of the best documented findings in the literature on perceptions of inequality is that people at both ends of the income distribution tend to place themselves near the middle. This systematic bias in self-perceptions is referred to as positioning bias, i.e., the difference between one’s subjective and objective position in the income distribution. Simply put, people at the top of the income distribution tend to exhibit negative bias (i.e., underestimate their position), while those at the bottom tend to exhibit positive bias (i.e., overestimate their position). This pattern holds within different countries (Bublitz, 2022), within the EU (Sattler-Bublitz et al., 2024), and internationally (Becker, 2023; Fehr et al., 2022; Nair, 2018).

Recent research highlights that there is substantial cross-national variation in positioning bias (Bublitz, 2022) and that this bias is consequential for policy preferences (e.g., Cruces et al., 2013; Engelhardt and Wagener, 2018; Karadja et al., 2016). This is because positioning bias undermines the frequent assumption that preference formation follows self-interest while being “fully informed” (e.g., Meltzer and Richard, 1981, p. 924) about their position within the income distribution. Following this logic, people may hold preferences that do not reflect their objective position due to the “‘technical’ failure” (Luebker, 2018, p. 22) of over- or underestimating their income position.

In this article, we examine an argument that is often put forward to explain cross-national differences in “misperceived self-interest,” but has received little empirical scrutiny. Specifically, the argument suggests that objective income inequality exacerbates existing perceptual biases by segregating the life worlds of the rich and poor, thereby restricting their exposure to inequality—i.e., information about the full range of incomes—in everyday life (e.g., Hing et al., 2019; Mijs, 2021; Willis et al., 2022). In countries with higher levels of income inequality, people may therefore draw on more homogeneous “subjective samples” (Evans and Kelley, 2004) to infer their own position within society, leading to increased positioning bias among the population.

Therefore, we address the question of whether people are more likely to be biased in their self-perception under conditions of high-income inequality. Furthermore, based on the literature on network segregation, we explore whether the effect of inequality varies across income groups and by type of bias. To this end, we combine cross-national data on 29 countries surveyed within the latest ISSP Social Inequality module (ISSP Research Group, 2022) with country-level data on objective inequality and employ multilevel modelling.

The remainder of the article is structured as follows: In Section 2, we elaborate on the theoretical mechanism that links objective income inequality to increased positioning bias. This entails a discussion of the importance of exposure to inequality through everyday experiences for how individuals perceive and locate themselves in society, and how objective inequality might condition exposure to inequality by segregating life worlds (e.g., Mijs, 2021). Section 3 then provides a description of the data and methods used. Section 4 presents the results, which are finally discussed and put into the context of past and future research in Section 5.

## 2 Previous literature

### 2.1 Exposure to inequality and positioning bias

Existing literature on inequality perceptions produces two recurring findings: first, subjective inequality perceptions are systematically biased with poorer (richer) people consistently overestimating (underestimating) their relative income position, i.e., positioning bias; second, the overall extent of inequality is generally underestimated (e.g., Bublitz, 2022; Cruces et al., 2013; Dawtry et al., 2015; Engelhardt and Wagener, 2018; Hauser and Norton, 2017).

To explain this, previous research has emphasized the importance of reference groups and local contexts for the formation of inequality perceptions. This seems intuitive, since in order to locate themselves in the income distribution or gauge inequality in general, respondents’ answers are based on an *availability heuristic* (Tversky and Kahneman, 1974). In other words, they draw on information that is readily available to them—and what information that is depends in part on the information that people are exposed to in their personal networks and local environments.

However, the information provided in these contexts is likely to be biased since local reference groups are not random samples of the population. Instead, they are the outcome of homophilic network formation, meaning that people are more likely to be exposed to individuals similar to themselves (Mcpherson et al., 2001; cf. Schulz et al., 2022). Therefore, “income-based homophily” (Mijs and Usmani, 2024, p. 3) leads people to move in contexts which disproportionately expose them to information about incomes close to their own. As a result, they obtain information from unrepresentative “subjective samples” (Evans and Kelley, 2004; Kelley and Evans, 1995), leading to biased inferences about their own position and aggregate inequality (Cruces et al., 2013; Knell and Stix, 2020; Schulz et al., 2022). Simply put, “[i]dividuals may over-generalize from their immediate reference group, thus making biased inferences” (Gimpelson and Treisman, 2018, p. 50). Further, they “may also—wrongly—take the range they observe in day-to-day interactions as a measure of the range nationwide” (Gimpelson and Treisman, 2018).

This is supported empirically by Dawtry et al. (2015), who show that wealthier respondents also report higher mean incomes for their social circles, and in turn inferred the rest of the population to be more affluent. In an Argentinian survey experiment, Cruces et al. (2013) show that the relative income position within respondents’ neighborhoods influences where they locate themselves in the national income distribution. In addition, having contact with a diverse set of social classes reduces positioning bias (Cruces et al., 2013). Minkoff and Lyons (2019) find that respondents living in more income diverse neighborhoods—i.e., contexts that expose people to information about a broader range of incomes –, are also more likely to perceive greater inequality. Taken together, these results demonstrate how local contexts can expose individuals to more or less biased informational samples, which are then used to infer their objective position at the national level.

### 2.2 The role of income inequality in conditioning exposure to inequality

Building on the preceding considerations, previous research has suggested that objective income inequality might reinforce perceptual biases (e.g., Hing et al., 2019; Mijs, 2021; Willis et al., 2022). The theoretical mechanism behind this is that income inequality serves as a proxy for socio-economic segregation and thus “conditions their exposure to inequality” (Mijs, 2021, p. 30) in everyday life. However, as outlined above, exposure to inequality—that is, information about a broad range of incomes within a person’s subjective sample of the social world—is crucial in order to form more accurate perceptions of society and one’s place within it (Mijs and Usmani, 2024; Schulz et al., 2022).

While this kind of argument has received considerable attention in some form or another,^1^ only few studies directly investigate how inequality shapes exposure to inequality. More attention has been paid to the relationship between income inequality and spatial segregation, which is then argued to reduce exposure to inequality.

In the United States, income inequality has been linked to increasing spatial segregation between income groups in the US (Reardon et al., 2018; Reardon and Bischoff, 2011). A similar trend, albeit to a more modest extent, has been observed across Europe (Marcińczak et al., 2015; Musterd et al., 2017). In addition, Nieuwenhuis et al. (2020) show that a combination of high segregation and inequality further intensifies socio-economic segregation by reducing the chances for upward spatial mobility, i.e., moving to less deprived neighborhoods. Mijs and Roe (2021) document that rising income inequality in the United States has been accompanied by increasing socio-economic segregation within social networks, schools, workplaces and neighborhoods. However, these institutions act as “inferential spaces” that determine the range of information to which people are exposed (Mijs, 2018). Therefore, more unequal societies are “likely to further limit people’s ability to make accurate inferences” (Mijs and Usmani, 2024, p. 14) due to producing more segregated social worlds that limit exposure to inequality in these formative institutions.

Taken together, this lends support to the idea that “income inequality shapes citizens’ interactions across socioeconomic lines and conditions their exposure to inequality “(Mijs, 2021, p. 30), creating a “feedback loop, where more inequality paradoxically leads them to experience less of it” (Mijs, 2021, p. 31). Based on this, we hypothesize that high-inequality contexts reduce individuals’ ability to accurately infer their relative position within the income distribution.

### 2.3 Differences between income groups

So far, we have assumed that exposure to inequality (i.e., access to information) and the effect of income inequality on positioning bias are the same across income groups. Hence, higher levels of inequality should be associated with more pronounced positioning bias due to an equal tendency for underestimation at the top and overestimation at the bottom of the income distribution.

However, research on social networks and social capital indicates that exposure to inequality might be stratified by income in the first place. For example, Lindh and Andersson (2024) find that individuals in higher class positions have more extensive ties to people in lower-middle- and working-class occupations. In contrast, individuals in lower class positions maintain fewer ties to the rest of society (Lindh and Andersson, 2024). In general, individuals in higher income positions tend to come into contact with people from both ends of the social spectrum more frequently, whereas people with lower incomes typically have lower levels of socioeconomic diversity within their social circles (Otero et al., 2021). This is corroborated by Pichler and Wallace (2009, p. 330), who demonstrate that “higher classes meet different people more often whereas working class people tend to have a smaller circle of social connections.”

Therefore, high-income individuals tend to have access to a more diverse and extensive sample of incomes. Against this backdrop, we expect that people at the top of the distribution are more accurate in their self-perceptions. Moreover, income inequality might have heterogeneous effects across income groups with regard to the theoretical mechanism that links inequality to positioning bias. In line with the argument linking inequality to socio-economic segregation, Pichler and Wallace (2009) argue that “the distance between social classes is larger in more unequal countries,” suggesting that “where social stratification is strongest [.] there will be less extensive social networks, because these are more likely to be limited to particular social classes.” This is supported in their analysis by finding that income inequality increases the previously established class differences in the participation in formal networks. Along these lines, Lancee and Van de Werfhorst (2012) provide evidence that low-income individuals in high-inequality countries are less likely to participate in civic and social life. Regarding network composition, Otero et al. (2024) find that income inequality moderates the relationship between socioeconomic status and network diversity, suggesting that low-status people are more restricted in their networks when living in high inequality countries. Following this strand of literature thus leads to the conclusion that higher levels of income inequality predominantly reduce exposure to inequality for poorer individuals because their networks represent more homogeneous samples that especially lack information on higher incomes.

On the other hand, different studies provide evidence for a U-shaped pattern of segregation, meaning that income groups at both ends of the income distribution “have networks that are mostly homogeneous” (Otero et al., 2021, p. 670). In a simulation study, Schulz et al. (2022) argue that inequality reinforces a skewed u-shaped segregation pattern. This suggests that particularly those in the richest decile become more segregated from the rest, creating “echo chambers for the richest whose information sets do not cover the poorer population at all” (Schulz et al., 2022, p. 317). This is further corroborated by Leo et al. (2016) who draw on communication and location data of Mexican mobile phone users. They find evidence for strong income-based homophily in connectedness and living environment, which is most pronounced among the richest, suggesting the existence of “tightly connected rich clubs “(Leo et al., 2016, p. 6). This suggests that the richest reduce their exposure to inequality by withdrawing into more exclusive social settings which are not accessible for the poorest of society.

Therefore, while previous research indicates that higher income positions are related to broader and more diverse networks, it remains unclear whether high inequality reduces exposure to inequality disproportionately for those at the top, the bottom, or both. Thus, while we do not derive explicit expectations about who is affected the most, the initial argument that inequality produces more homogeneous reference groups implies that higher inequality intensifies the dominant biases at each end of the income distribution. We therefore hypothesize that higher inequality is related to increased underestimation among the affluent and overestimation among the poor.

## 3 Data and methods

To empirically investigate our research question, we draw on the latest version of the Social Inequality module of the International Social Survey Programme, which was conducted in 2019. This allows us to examine the relationship between income inequality, income position, and positioning bias using cross-national, individual-level survey data from 29 countries.

Our main dependent variable is *positioning bias.* To measure this, we construct a variable based on country-specific equivalized (square root scale) household income deciles and a survey item used for measuring subjective social status on a 10-point scale. For our analysis, we use a binary measure of positioning bias that indicates whether or not a respondent is biased in their self-positioning. Following previous studies, we classify respondents as biased when their reported subjective social status on the 10-point ladder differs by two or more points from their income decile (cf. Bublitz, 2022; Cruces et al., 2013). This binary measure allows us to investigate our main question, i.e., whether individuals in more unequal countries are more likely to exhibit positioning bias. In addition, we construct a categorical measure that further differentiates the type of bias, i.e., whether individuals are unbiased, overestimate or underestimate their position. This variable is used to provide a more in-depth analysis of the type of positioning bias exhibited by different income groups across levels of inequality.

We are aware that equating subjective status with income position is problematic, given that subjective status is multidimensional, incorporating factors such as educational attainment and occupational status (Galvan et al., 2023). Furthermore, we have to assume that the two scales are comparable, despite this potentially not being the case (cf. Anderson, 2025). These problems are common (cf. Bargain et al., 2025; Condon and Wichowsky, 2020), as most surveys include a broader measure of subjective status, but not the respondents’ perceived income position. We try to address the former problem by providing additional robustness checks, replacing income with education, occupational prestige, or an index combining all three (see Section 4.3).

The main independent variable at the country-level is *income inequality*. To measure this, we use the top 10% income share of disposable income from the World Inequality Database (wid.world). We chose this measure as previous research has shown that segregation is particularly driven by the affluent (Reardon and Bischoff, 2011). Thus, their income share could be particularly important with regard to the proposed theoretical mechanism. The commonly used Gini index is rather insensitive to changes at the tails of the income distribution (Osberg, 2017). However, we provide robustness checks using the Gini index of disposable income (see Section 4.3).

At the individual level, we use a categorical measure of income to explore whether the effect of inequality varies across income groups. Thus, we group respondents into country-specific income tertiles.

Moreover, we use a set of control variables to account for possible confounders between the variables of interest. On the country-level, we control for (PPP-adjusted) GDP per capita in $1,000 (Feenstra et al., 2015) to account for differences in general economic prosperity between countries (Evans and Kelley, 2004). At the individual level, we account for compositional effects by controlling for years of full-time education, occupational prestige, age and sex (men [ref.], women).

Given the hierarchical structure of the data, we use multilevel regression models. Multilevel models account for clustering of observations within countries and allow us to model between-country variance by letting the intercept $\beta_{0}$ vary across countries by adding country-specific random effects $u_{j}$ (Snijders and Bosker, 2012). Because our main dependent variable is binary, we use a multilevel logistic regression model to predict the probability that individual i in country j exhibits positioning bias $\left(Y_{\mathrm{ji}}=1\right)$ , with individuals i(1,2,…,n) clustered in countries j(1,2,…,29) . Thus, the baseline model is given by Equation 1.


$$\text{logit}(\mathrm{Pr}(Y_{\mathrm{ji}}=1))=\beta_{0}+\beta X_{\mathrm{ji}}+\gamma Z_{j}+u_{j}$$ (1)

where $\beta X_{\mathrm{ji}}$ represents a vector of individual-level predictors and their fixed effects, and $\gamma Z_{j}$ a vector of country-level predictors and their fixed effects (see above). Building on this baseline model, we add a cross-level interaction term between income inequality and income tertiles, along with random slopes for the income groups (Heisig and Schaeffer, 2019). All models are estimated using the weights provided by the ISSP,^2^ as well as clustered standard errors.

Furthermore, to provide a more detailed examination of positioning bias, distinguishing between underestimation, no bias, and overestimation, we follow Bublitz (2022, p. 442). by extending the analysis to a (multilevel) multinomial logistic regression model (StataCorp, 2023).^3^ For the multinomial model, we specify $Y_{\mathrm{ji}}=k$ (where k=1 for no bias, k=2 for underestimation, and k=3 for overestimation, with k=1 as the reference category). Because the coefficients in these models are expressed as (multinomial) log-odds relative to the reference category, we focus on presenting predicted probabilities for substantive conclusions. All presented results are based on the same sample of 29 countries and 31,368 individuals, with an average of 1,082 respondents per country.

## 4 Results

### 4.1 Descriptive results

**Figure 1:**
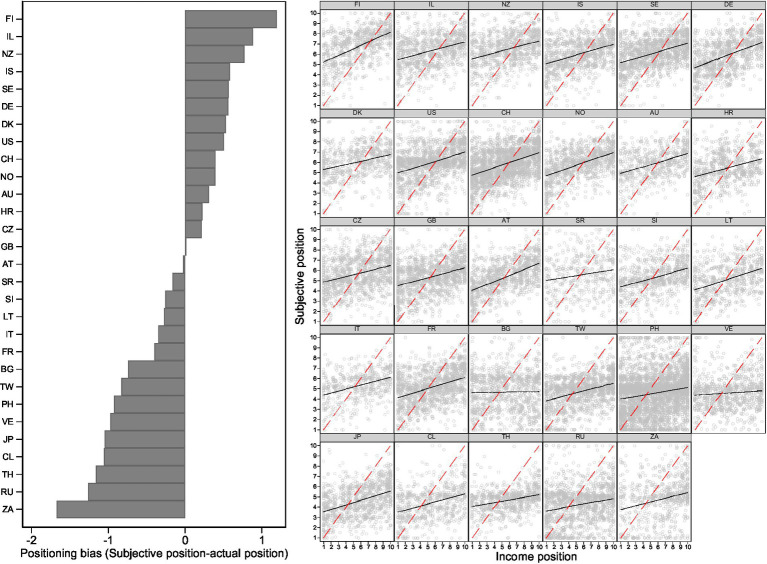
Average positioning bias by country and actual income position.

Before moving on to our main analyses, Figure 1 provides a first overview of the direction and degree of bias in each country. The left-hand panel shows which type of bias is most common in each country. Positive (negative) values indicate that people, on average, report a higher (lower) subjective position than their objective position. In Finland, for example, people tend to overestimate their position by one decile, while South Africa has the largest negative bias, underestimating by close to two deciles. In Austria and Great Britain, the subjective position is, on average, in line with the objective position amounting to a positioning bias of close to zero in both countries. This is also reflected on the right-hand side of Figure 1. The dashed 45-degree line depicts how subjective and objective positions align in the case of no bias (cf. Bublitz, 2022, p. 443f). The points indicate how individuals’ subjective positions are distributed over their income position, while the intercept and slope of the black regression lines show how each decile deviates from the 45° line. Austria combines a low intercept with a steep slope, suggesting a comparatively small and equal degree of overestimation at the bottom and underestimation at the top. In contrast, countries like Bulgaria and Venezuela show a rather flat slope, suggesting that the subjective position hardly varies with the actual income position. In sum, this figure shows that there is considerable variation in the direction and degree of bias between countries.

**Figure 2:**
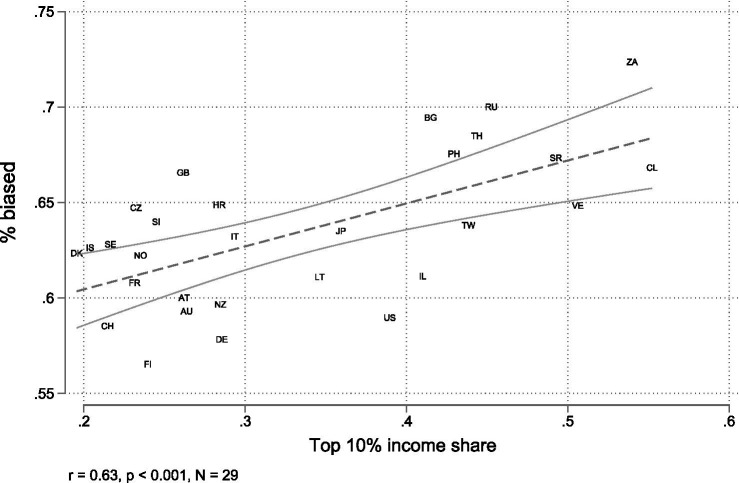
Bivariate macro-level relationship between the share of biased respondents and the top 10% share of disposable income.

Figure 2 depicts the relationship between the top 10% income share and the percentage of people who are biased across 29 countries. Each point represents a country and the regression line indicates a positive correlation (*r* = 0.6, *p* < 0.001). This means that higher levels of income inequality at the country-level are associated with a greater share of people who are biased, i.e., miss their objective rank in the income distribution by at least two deciles. At the extremes, we observe countries such as Chile (CL) and South Africa (ZA), which exhibit high income concentration and a high proportion of biased respondents, while countries like Finland (FI) and Switzerland (CH) show low values with regard to both.

This bivariate macro-level relationship is a first indication that higher levels of income inequality are related to more pronounced positioning bias. However, it also hides the substantial heterogeneity in the direction of bias between income groups and countries that is presented in Figure 1.

### 4.2 Multilevel analysis

To test whether more unequal contexts amplify prevalent biases, we first use multilevel logistic regression to examine the probability that an individual exhibits positioning bias. Table 1 reports the regression results. Model 1 includes only the top 10% income share at the country-level. This model reproduces the results observed in Figure 2, providing preliminary evidence for our hypothesis by indicating that higher income inequality is associated with an increase in the log-odds of positioning bias. The addition of individual-level variables in M2 does not significantly alter this effect. Higher occupational prestige is related to decreased positioning bias, while age and gender show no significant effects.

**Table 1:** Multilevel logistic regression of positioning bias.

	M1	M2	M3	M4
	b/se	b/se	b/se	b/se
Top 10% income share	1.098^***^	1.126^***^	0.462^+^	−3.224^***^
	(0.202)	(0.224)	(0.261)	(0.691)
Income tertile (ref. bottom)				
Middle tertile		−1.361^***^	−1.361^***^	−2.832^***^
		(0.150)	(0.150)	(0.258)
Top tertile		0.274	0.274	−2.328^***^
		(0.264)	(0.264)	(0.512)
Education		0.001	0.001	−0.002
		(0.003)	(0.004)	(0.003)
SIOPS		−0.003^*^	−0.003^*^	0.000
		(0.001)	(0.001)	(0.001)
Age		0.001	0.001	0.001
		(0.001)	(0.001)	(0.001)
Female (ref. male)		0.018	0.018	0.011
		(0.030)	(0.030)	(0.027)
GDP per capita			−0.005^***^	−0.005^*^
			(0.002)	(0.002)
Middle tertile # Top 10% income share				4.138^***^
				(0.786)
Top tertile # Top 10% income share				7.954^***^
				(1.568)
Constant	0.191^**^	0.656^***^	1.086^***^	2.303^***^
	(0.071)	(0.188)	(0.258)	(0.296)
*Random effects*				
var (country)	0.013^***^	0.015^***^	0.011^***^	0.198^**^
	(0.003)	(0.003)	(0.003)	(0.073)
var (middle tertile)				0.251^**^
				(0.086)
var (top tertile)				1.125^***^
				(0.304)
cov (middle tertile, top tertile)				0.435^**^
				(0.165)
cov (middle tertile, country)				−0.208^**^
				(0.080)
cov (top tertile, country)				−0.431^**^
				(0.145)
N_indiv_	31,368	31,368	31,368	31,368
N_country_	29	29	29	29

+*p* < 0.10; *p < 0.05; **p < 0.01; ***p < 0.001; weighted; cluster-robust standard errors in parentheses.

Source: Author’s own calculations, ISSP 2019.

Looking at the coefficients for the middle and upper tertile in reference to the lowest tertile, it becomes apparent that only the middle tertile is significantly less biased. Thus, this result contradicts the expectation that high-income individuals are less biased in their self-positioning than low-income individuals due to their access to a more diverse and extensive sample of incomes. M3 adds GDP per capita as a control variable. GDP per capita is negatively associated with positioning bias, indicating that individuals living in richer countries are less likely to be biased. Controlling for GDP per capita reduces the direct effect of income inequality and pushes its significance above the 5% level (*p* = 0.077).^4^ This suggests that the direct relationship between inequality and positioning bias might be partially confounded by general economic prosperity.

**Figure 3:**
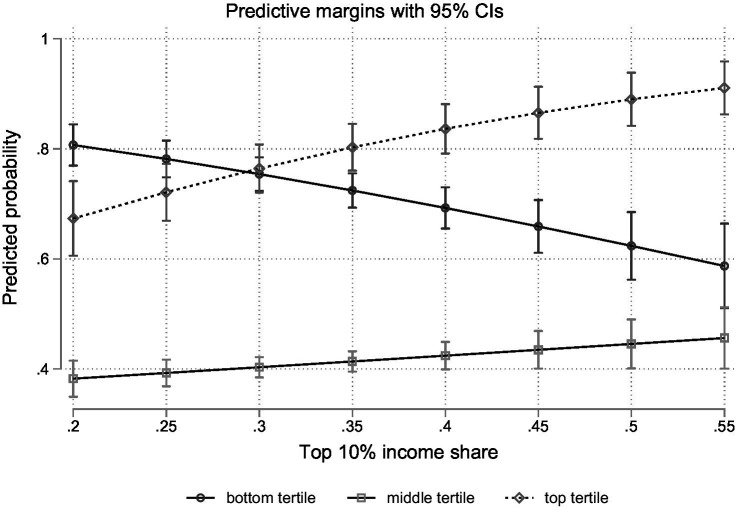
Predicted probabilities of positioning bias by income group and across inequality levels (based on M4, Table 1).

However, looking at these aggregate effects might mask important differences between income groups. To explore whether the effect of income inequality on positioning bias varies across income groups, Model 4 introduces cross-level interactions between income inequality and income tertiles. These interactions test whether inequality disproportionately amplifies underestimation or overestimation for specific socioeconomic groups, as discussed in Section 2.3. All main and interaction effects are significant (*p* < 0.001; see M4). To facilitate the substantive interpretation of the cross-level interaction effects, Figure 3 presents the predicted probabilities of each income tertile exhibiting positioning bias across the range of the top 10% income share.

The figure shows that higher levels of income inequality are only associated with a higher probability of positioning bias among those in middle- and high-income positions. For example, moving from a top 10% income share of 25 to 45% (i.e., moving roughly from one standard deviation below the mean to one above) increases the probability of positioning bias by 4.2% points among middle-income individuals and by 14.2% points among high-income individuals. This amounts to respective probabilities of 43 and 87% of exhibiting bias. Conversely, individuals in low-income positions are predicted to be increasingly accurate in contexts of high inequality. For them, the probability of positioning bias decreases by circa 12.3% points across the same range. Thus, in egalitarian countries, positioning bias seems to be more prevalent among those in low-income positions, while in high-inequality contexts particularly those at the top are more inaccurate in their self-perceptions. Strikingly, where the top 10% income share is highest, middle- and low-income individuals are very close in their probability of exhibiting bias.

**Figure 4:**
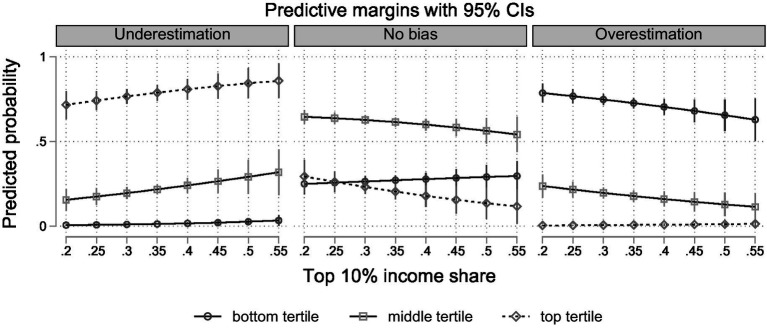
Predicted probabilities of overestimation, underestimation and no bias by income group and across inequality levels (based on M8 in Table OA1).

While it is reasonable to assume that these effects are driven by decreased overestimation at the bottom and increased underestimation at the top, these results do not tell us which type of bias becomes more likely among the middle. As discussed in Section 3, we use multilevel multinomial logistic models (StataCorp, 2023) to provide further evidence regarding the high- and low-income positions, and explore which type of bias is more prevalent among the middle. The results again indicate that higher income inequality is associated with a higher (lower) probability of underestimation (overestimation) in reference to no bias, especially before controlling for GDP per capita (see M5-M7 in Tab A1). In M8, we again add interaction effects between income inequality and income groups (see Table OA1). In this model, the main effects of income inequality are significant for underestimation and marginally significant for overestimation, while the interaction term is only significant for the top income tertile with regard to overestimation. However, substantive conclusions about interaction effects can only be made by looking at predicted probabilities, especially in non-linear models (cf. Mize, 2019). Thus, we again display the results graphically to facilitate interpretation of the cross-level interaction effects. Figure 4 presents the predicted probabilities for each of the possible outcomes by income group and across inequality levels.

The results confirm that the affluent are more likely to be biased due to underestimating their position when inequality is high. Conversely, the probability of overestimation decreases across higher levels of inequality among the less well-off. In the middle of the income distribution, over- and underestimation have about the same probability when inequality is average. However, different types of bias are more likely in high versus low inequality contexts: Under high inequality, those in the middle are likely to underestimate their position, while they tend more toward overestimation in egalitarian contexts. This reveals that the increased probability of bias found in M4 results from an increased tendency to overestimate. Hence, these patterns reflect that higher inequality is associated with lower subjective status, relative to the objective income position, across all income groups.

In summary, the results presented provide evidence to support the conclusion that income inequality is related to increased positioning bias at the aggregate level. That is, individuals in high-inequality contexts are, on average, more likely to be biased. However, this conclusion requires substantial qualification: Rather than exacerbating dominant biases uniformly across income groups, as is often assumed, significant differences emerge between them. While higher inequality is associated with an increased tendency to underestimate at the top, and in the middle, those at the bottom are more likely to hold more accurate self-perceptions, reflecting lower subjective status among all income groups.

### 4.3 Robustness

To ensure the robustness of our results, we conduct a number of robustness checks for our main models (M1–M4) that address the operationalisation of our main dependent and independent variables. First, we re-run the models using the disposable Gini as an alternative inequality measure. The results are substantively the same (see Supplementary Table OA2 and Figure OA1). Second, due to the limitation that the ISSP data only provides information on the subjective status of individuals, instead of the subjective income position, our operationalisation might not adequately account for the multidimensionality of subjective status. As subjective status incorporates factors such as educational attainment and occupational status (Galvan et al., 2023), we provide additional analyses in which the income position is replaced by education, occupational prestige, or an index combining all three.

**Figure 5:**
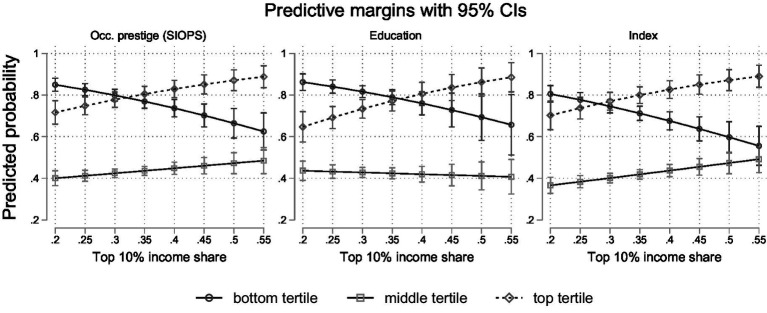
Predicted probabilities of positioning bias by income group and across inequality levels (based on Table OA2).

While the main effect of inequality is only significant for the status index, the interaction results are substantively the same regardless of the operationalisation of the dependent variable, suggesting that the observed relationship between income inequality, income position, and positioning bias is robust across different dimensions of socioeconomic status (see Figure 5 and Supplementary Table OA3). Substantively, this means that higher inequality is associated with decreased subjective status relative to the respondent’s objective status across status groups and status indicators. The only difference emerges when only considering education. Here, those in the middle tertile remain roughly equally likely to miss their objective position across all levels of inequality.

To test whether income inequality moderates the relationship between income and positioning bias while accounting for overall economic prosperity, we re-estimate M4 and M8 while simultaneously including cross-level interactions between GDP per capita and income tertiles (see Supplementary Tables OA4 and OA5). When these cross-level interactions are added to the multilevel logit model, the interactions with inequality attenuate and turn insignificant. However, given the high correlation between GDP and income inequality (*r* = 0.75) and the limited country sample, this might introduce problems of collinearity. Using log-transformed GDP reduces the correlation (*r* = 0.6) and produces results that remain very close to those presented in M4. Adding the cross-level interactions to the multilevel multinomial models reveals that higher GDP shows the reverse pattern of inequality, increasing subjective status relative to income position. Thus, higher GDP is associated with a lower probability of overestimation among the top and middle tertile, while increasing overestimation among the bottom tertile. Based on this, we acknowledge that our research design does not allow us to disentangle these effects. At the same time, although significance and effect sizes vary, the qualitative pattern found in our main analysis remains robust across specifications (see Supplementary Figure OA2). Further, since Figure 2 shows that two broad country clusters emerge, we address potential concerns about regional clustering, we conduct robustness checks using different country classifications (see Supplementary Figure OA4). First, we add dummies indicating whether a country is early or late industrialized (Castillo et al., 2023 based on Ishida, 2008), developed or developing according to the UN, Global North or Global South, and above or below the median GDP per capita. Then, we test whether the main effect of inequality is driven by one subset of these countries by including interactions between regional dummies and the top 10% income share. When controlling for the early/late industrialization classification or for countries below/above the sample mean in terms of GDP per capita, the main effect remains similar in size and significance. Controlling for the Global North/South classification reduces the effect by almost half, rendering it insignificant. Adding the UN development dummy increases the effect, but still renders it insignificant, likely due to limited statistical power given that the sample comprises only seven developing countries. However, allowing the effect to vary by region reveals substantial heterogeneity: the coefficient is larger and more significant (*p* < 0.05) in late industrialized and low GDP contexts, and similar to the baseline model in the Global South. On the other hand, the effect is insignificant and close to zero or even negative in above median GDP, early industrialized or Global North contexts. Thus, with the exception of the UN classification, the results suggest that the relationship is consistently stronger and more precisely estimated in less developed contexts. Further analysis examining the interaction between inequality and income position within high- and low-GDP country subsamples separately (see Supplementary Table OA6) confirms this pattern, with cross-level interactions remaining pronounced and significant in low-GDP countries even when cross-level interactions between GDP per capita and income tertiles are included simultaneously.

## 5 Discussion

Biased subjective inequality perceptions have gained a lot of attention in explaining the apparent lack of response to actual inequality (Hing et al., 2019; Trump, 2023; but see Wiesner, 2025). Previous research has firmly established that individuals at either end of the income distribution tend to be biased toward locating themselves in the middle. As a consequence, preference formation may not actually reflect people’s self-interest while being “fully informed” (e.g., Meltzer and Richard, 1981, p. 924), as often assumed in rational choice frameworks. Thus, people may hold policy preferences that do not reflect their objective position due to the “‘technical’ failure” (Luebker, 2018, p. 22) of over- or underestimating their income position.

Against this backdrop, this article set out to examine the question of whether higher levels of income inequality are related to increased positioning bias across countries. An argument that is often put forward suggests that individuals draw on readily available information from local contexts to infer their position at the national level. However, these “subjective samples” (Evans and Kelley, 2004) disproportionately expose them to incomes similar to their own due to “income-based homophily” (Mijs and Usmani, 2024, p. 3; Schulz et al., 2022), leading them to make biased inferences about their own position at the national level (Cruces et al., 2013). Income inequality is then argued to exacerbate existing perceptual biases due to its link to socio-economic segregation and, consequently, reduced exposure to inequality—i.e., even more homogeneous subjective samples—in everyday life (e.g., Mijs, 2021).

Based on comparative survey data from 29 countries, our results provide evidence that income inequality is indeed related to increased perceptual biases in the form of positioning bias. However, not in a uniform manner across income groups; instead, the association is predominantly driven by increased underestimation among high-income individuals, while those in low-income positions tend to be more accurate in their self-perceptions under high inequality. Among those in the middle, the dominant bias shifts from overestimation in egalitarian contexts to underestimation in contexts of high inequality. These patterns reflect a downward shift in subjective status, relative to respondents’ objective income ranks across all income groups.

These results are not in line with the hypothesis that inequality increases positioning bias at both ends of the income distribution. This may indicate a scenario in which higher income inequality reinforces a skewed U-shaped pattern of segregation, with those at the top reducing their exposure to lower incomes (Schulz et al., 2022; Leo et al., 2016), while the rest is becoming more aware of their relatively disadvantaged position due to more pronounced stratification. However, this interpretation rests on the strong assumption that income inequality serves as a proxy for socio-economic segregation, and that this segregation then leads individuals at the top to be less exposed to lower incomes. Another perspective suggests that income inequality intensifies feelings of relative deprivation by increasing income differences as well as the salience of very high incomes against which people compare themselves, leading to lower subjective status across the income distribution (Schneider, 2019). Furthermore, our robustness checks show the reverse pattern for GDP, indicating that higher GDP is associated with less underestimation at the top and more overestimation at the bottom, reflecting higher subjective status across the income distribution. Moreover, we find that the effect of inequality is more pronounced and robust in some regional contexts than in others, suggesting that income inequality depresses subjective status across the income distribution particularly in less economically developed contexts where economic disparities may be more visible and salient in everyday life.

Previous literature proposed that inequality might be self-reinforcing because biased perceptions of inequality produce a lack of concern and even undermine support for redistribution (e.g., Mijs, 2021; Windsteiger, 2022). From a rational choice perspective, this would be the case if inequality leads “poor people to underestimate what they can gain from redistribution and therefore to show less support for redistribution than if they would have perfect knowledge of the income distribution” (Windsteiger, 2022, p. 10). While the literature is inconclusive on the effects of correcting misperceptions in the first place, some studies show the expected patterns: information about overestimation increases support for redistribution (Cruces et al., 2013), whereas discovering an advantage tends to reduce it (Bublitz, 2022; Engelhardt and Wagener, 2018).

In light of our findings, is it plausible that cross-national differences in support for redistribution stem from the “technical failure” (Luebker, 2018, p. 22) of misperceived self-interest? Since our results suggest that high inequality fosters underestimation among those in the middle and particularly among the well-off, while those at the bottom are more aware of their disadvantaged position, correcting misperceptions might not increase the demand for redistribution in high inequality countries. In fact, correcting people’s self-perceptions might even reinforce existing attitudinal divides between income groups (cf. Wiesner, 2025) as more people at the top in high-inequality countries would recognise their advantaged position, and, conversely, those at the bottom in more equal countries their disadvantaged one. Nevertheless, this remains speculative and open to further empirical investigation.

Finally, we have to note that our results are subject to several limitations. The item measuring the subjective position asks respondents about their subjective status rather than their perceived income position. Although the relative income position is an important determinant, other factors such as educational attainment or the recognition one experiences also determine subjective status (Galvan et al., 2023; see Anderson, 2025 for a recent critique of the subjective status measure). We have tried to address this issue by showing that our findings replicate across different status measures. However, previous studies argued that positioning oneself in the middle can be a moral claim of ordinariness (Irwin, 2018; Savage et al., 2001). Along these lines, Sherman (2017, p. 232) concludes that “the middle is symbolically available to everyone, even those at the very top of the income distribution.” Therefore, positioning bias may not only be the result of misinformation, but also “part of a quest to appear ‘normal’” (Savage et al., 2001, p. 887). A further limitation is that our study can only address associations between income inequality and positioning bias. While our results do not lend support to the idea that inequality is related to increased bias across income groups, we were not able to directly test the proposed mechanism of socio-economic segregation and reduced exposure. Future research could address this by, for example, incorporating direct measures of segregation or network data, ideally over time.

## Data Availability

Publicly available datasets were analyzed in this study. This data can be found here: doi: 10.4232/1.14009.

## Footnotes

^1^E.g., Condon and Wichowsky (2020, pp. 6, 21 f); García-Castro et al. (2022, p. 287); Hing et al. (2019, p. 110); Kelly and Moore-Clingenpeel (2012, p. 143); Mijs and Usmani (2024); Mijs (2021, p. 31); Mijs and Roe (2021, p. 11); Minkoff and Lyons (2019, p. 2); Willis et al. (2022, p. 304).

^2^Depending on the country, the provided weighting variable includes design weights, post-stratification weights, or no weights. Countries that use household-based samples are required to provide design weights (see ISSP Research Group, 2022).

^3^These models do not include random slopes for the lower-level variables involved in the cross-level interaction due to convergence issues.

^4^Using log-transformed GDP per capita instead does not alter the size or significance of the coefficient (see M21 in the Supplementary material).
