# Supplementary material for: Knowing one’s place? The role of income inequality in shaping positioning bias across 29 countries
Source: Front Sociol. 2026 Jan 6;10:1617014. doi: 10.3389/fsoc.2025.1617014 (PMC12815798; doi:10.3389/fsoc.2025.1617014)
Supplement: Supplementary file 1 [file Supplementary_file_1.docx]

***Supplementary Material for Knowing One's Place? The Role of Income Inequality in Shaping Positioning Bias Across 29 Countries***

Table OA1. Multilevel multinomial logistic regression of positioning bias

|  | M5 | | M6 | | M7 | | M8 | |
| --- | --- | --- | --- | --- | --- | --- | --- | --- |
| no bias | Under | Over | Under | Over | Under | Over | Under | Over |
| (ref.) | b/se | b/se | b/se | b/se | b/se | b/se | b/se | b/se |
| Top 10% share | 2.623^***^ | -0.848^**^ | 4.527^***^ | -2.361^***^ | 2.666^+^ | -1.996^+^ | 4.399^*^ | -2.128^+^ |
|  | (0.426) | (0.315) | (0.834) | (0.672) | (1.501) | (1.129) | (1.974) | (1.129) |
| GDP per capita | |  |  |  | -0.015^*^ | 0.003 | -0.000^*^ | 0.000 |
|  |  |  |  |  | (0.000) | (0.006) | (0.000) | (0.000) |
| Income tertile (ref. bottom) | | |  |  |  |  |  |  |
| Middle tertile | |  | 2.297^***^ | -2.393^***^ | 2.296^***^ | -2.394^***^ | 3.059^***^ | -2.414^***^ |
|  |  |  | (0.155) | (0.074) | (0.155) | (0.074) | (0.619) | (0.219) |
| Top tertile |  |  | 4.931^***^ | -5.044^***^ | 4.930^***^ | -5.046^***^ | 5.568^***^ | -7.362^***^ |
|  |  |  | (0.168) | (0.305) | (0.169) | (0.305) | (0.561) | (0.644) |
| SIOPS |  |  | -0.008^**^ | 0.008^***^ | -0.008^**^ | 0.008^***^ | -0.008^**^ | 0.008^***^ |
|  |  |  | (0.003) | (0.002) | (0.003) | (0.002) | (0.003) | (0.002) |
| Education |  |  | -0.022^***^ | 0.016^***^ | -0.022^***^ | 0.016^***^ | -0.022^***^ | 0.015^***^ |
|  |  |  | (0.007) | (0.004) | (0.007) | (0.005) | (0.007) | (0.004) |
| Age |  |  | 0.001 | 0.002 | 0.001 | 0.002 | 0.001 | 0.002 |
|  |  |  | (0.002) | (0.002) | (0.002) | (0.002) | (0.002) | (0.002) |
| Female (ref. male) | |  | -0.008 | 0.039 | -0.009 | 0.039 | -0.008 | 0.040 |
|  |  |  | (0.053) | (0.029) | (0.053) | (0.029) | (0.053) | (0.029) |
| Middle tertile # Top 10% share | | |  |  |  |  | -1.882 | 0.058 |
|  |  |  |  |  |  |  | (1.568) | (0.756) |
| Top tertile # Top 10% share | | |  |  |  |  | -1.498 | 7.501^***^ |
|  |  |  |  |  |  |  | (1.462) | (2.204) |
| Constant | -1.010^***^ | 0.061 | -4.423^***^ | 1.204^***^ | -3.203^***^ | 0.955^+^ | -3.913^***^ | 1.008^+^ |
|  | (0.153) | (0.101) | (0.344) | (0.271) | (0.787) | (0.570) | (0.924) | (0.570) |
| *Random effects* | |  |  |  |  |  |  |  |
| var(over) | | 0.090^***^ |  | 0.312^***^ |  | 0.280^***^ |  | 0.279^***^ |
|  |  | (0.027) |  | (0.083) |  | (0.077) |  | (0.078) |
| var(under) | | 0.031^**^ |  | 0.162^***^ |  | 0.161^***^ |  | 0.160^***^ |
|  |  | (0.010) |  | (0.047) |  | (0.046) |  | (0.046) |
| cov(over, under) | | -0.033^**^ |  | -0.199^***^ |  | -0.193^***^ |  | -0.192^***^ |
|  |  | (0.013) |  | (0.058) |  | (0.054) |  | (0.055) |
| N_indiv_ | 31,368 |  | 31,368 |  | 31,368 |  | 31,368 |  |
| N_country_ | 29 |  | 29 |  | 29 |  | 29 |  |

*Notes: +P < 0.10; *P < 0.05; **P < 0.01; ***P < 0.001; weighted; cluster-robust standard errors in parentheses; under refers to underestimation; over refers to overestimation.*

*Source: Author's own calculations, ISSP 2019.*

Table OA2. Multilevel logistic regression of positioning bias (disposable Gini)

|  | M9 | M10 | M11 | M12 |
| --- | --- | --- | --- | --- |
|  | b/se | b/se | b/se | b/se |
| Disposable Gini | 0.821^***^ | 0.844^***^ | 0.274 | -2.588^**^ |
|  | (0.174) | (0.196) | (0.221) | (0.881) |
| Income tertile (ref. bottom) | |  |  |  |
| Middle tertile |  | -1.361^***^ | -1.362^***^ | -2.530^***^ |
|  |  | (0.150) | (0.150) | (0.454) |
| Top tertile |  | 0.274 | 0.272 | -1.768^*^ |
|  |  | (0.264) | (0.264) | (0.690) |
| SIOPS |  | -0.003^*^ | -0.003^*^ | -0.001 |
|  |  | (0.001) | (0.001) | (0.002) |
| Education |  | 0.001 | 0.001 | -0.002 |
|  |  | (0.003) | (0.004) | (0.003) |
| Age |  | 0.001 | 0.001 | 0.001 |
|  |  | (0.001) | (0.001) | (0.001) |
| Female (ref. male) |  | 0.017 | 0.017 | 0.020 |
|  |  | (0.030) | (0.030) | (0.028) |
| GDP per capita |  |  | -0.006^***^ | -0.006^***^ |
|  |  |  | (0.002) | (0.002) |
| Middle tertile # disposable Gini | |  |  | 3.100^**^ |
|  |  |  |  | (1.043) |
| Top tertile # disposable Gini | |  |  | 5.811^**^ |
|  |  |  |  | (1.770) |
| Constant | 0.234^**^ | 0.686^***^ | 1.142^***^ | 2.209^***^ |
|  | (0.072) | (0.206) | (0.267) | (0.489) |
| *Random effects* |  |  |  |  |
| var(country) | 0.014^***^ | 0.016^***^ | 0.011^***^ | 0.213 |
|  | (0.003) | (0.004) | (0.003) | (1.121) |
| var(middle tertile) |  |  |  | 0.261 |
|  |  |  |  | (1.225) |
| var(top tertile) |  |  |  | 1.185 |
|  |  |  |  | (5.042) |
| cov(middle tertile, top tertile) | |  |  | 0.425 |
|  |  |  |  | (2.487) |
| cov(middle tertile, country) | |  |  | -0.222 |
|  |  |  |  | (1.172) |
| cov(top tertile,country) | |  |  | -0.451 |
|  |  |  |  | (2.380) |
| N_indiv_ | 31,368 | 31,368 | 31,368 | 31,368 |
| N_country_ | 29 | 29 | 29 | 29 |

*Notes: +P < 0.10; *P < 0.05; **P < 0.01; ***P < 0.001; weighted; cluster-robust standard errors in parentheses.*

*Source: Author's own calculations, ISSP 2019.*

Figure OA1. Predicted probabilities of positioning bias by income group and across inequality levels (based on M12).

Table OA3. Multilevel logistic regression of positioning bias measured by different status variables

|  | M13 | M14 | M15 | M16 | M17 | M18 |
| --- | --- | --- | --- | --- | --- | --- |
|  | siops | siops | educ. | educ. | index | index |
|  | b/se | b/se | b/se | b/se | b/se | b/se |
| Top 10% income share | 0.376 | -3.619^***^ | -0.123 | -3.565^**^ | 0.691^*^ | -3.566^***^ |
|  | (0.261) | (0.758) | (0.897) | (1.348) | (0.277) | (0.810) |
| GDP per capita | -0.003^+^ | -0.006^*^ | 0.001 | 0.000 | -0.004^**^ | -0.005^*^ |
|  | (0.001) | (0.002) | (0.005) | (0.006) | (0.001) | (0.002) |
| Middle siops/educ/index tertile | -1.415^***^ | -3.156^***^ | -1.575^***^ | -2.859^***^ | -1.355^***^ | -3.082^***^ |
|  | (0.198) | (0.221) | (0.178) | (0.297) | (0.153) | (0.265) |
| Top siops/educ/index tertile | 0.201 | -2.230^***^ | -0.117 | -2.873^***^ | 0.308 | -2.026^***^ |
|  | (0.265) | (0.513) | (0.317) | (0.550) | (0.263) | (0.582) |
| Age | 0.001 | 0.001 | 0.002 | 0.004^*^ | -0.000 | 0.001 |
|  | (0.001) | (0.001) | (0.002) | (0.002) | (0.001) | (0.001) |
| Female (ref. male) | 0.092^*^ | 0.091^*^ | 0.013 | 0.022 | 0.081^**^ | 0.087^**^ |
|  | (0.043) | (0.042) | (0.032) | (0.027) | (0.027) | (0.027) |
| Status tertile (ref. bottom) |  |  |  |  |  |  |
| Middle tertile |  | 4.649^***^ |  | 3.198^**^ |  | 5.113^***^ |
|  |  | (0.749) |  | (1.095) |  | (0.733) |
| Top tertile |  | 6.993^***^ |  | 7.890^***^ |  | 7.209^***^ |
|  |  | (1.552) |  | (1.741) |  | (1.729) |
| Constant | 1.048^***^ | 2.688^***^ | 1.118^*^ | 2.462^***^ | 0.863^***^ | 2.344^***^ |
|  | (0.217) | (0.332) | (0.516) | (0.613) | (0.195) | (0.330) |
| *Random effects* |  |  |  |  |  |  |
| var(country) | 0.004 | 0.232^**^ | 0.041^**^ | 0.355^*^ | 0.009^*^ | 0.195^**^ |
|  | (0.003) | (0.090) | (0.015) | (0.178) | (0.004) | (0.060) |
| var(middle tertile) |  | 0.232^**^ |  | 0.606^*^ |  | 0.217^**^ |
|  |  | (0.090) |  | (0.239) |  | (0.079) |
| var(top tertile) |  | 1.029^***^ |  | 1.278^*^ |  | 1.063^***^ |
|  |  | (0.299) |  | (0.603) |  | (0.238) |
| cov(middle tertile, top tertile) |  | 0.380^*^ |  | 0.680^+^ |  | 0.372^**^ |
|  |  | (0.167) |  | (0.383) |  | (0.129) |
| cov(middle tertile, country) |  | -0.210^*^ |  | -0.418^*^ |  | -0.183^**^ |
|  |  | (0.089) |  | (0.206) |  | (0.068) |
| cov(top tertile, country) |  | -0.467^**^ |  | -0.587^+^ |  | -0.431^***^ |
|  |  | (0.163) |  | (0.322) |  | (0.115) |
| N_indiv_ | 31,368 | 31,368 | 31,368 | 31,368 | 31,368 | 31,368 |
| N_country_ | 29 | 29 | 29 | 29 | 29 | 29 |

*Notes: +P < 0.10; *P < 0.05; **P < 0.01; ***P < 0.001; weighted; cluster-robust standard errors in parentheses. Bottom, middle and top tertile refer to SIOPS, full-time years of education or the index of the latter and income respectively (see Section 4.3).*

*Source: Author's own calculations, ISSP 2019.*

Table OA4. Multilevel logistic regression of positioning bias using (log.) GDP and adding cross-level interactions with (log.) GDP

|  | M19 | M20 | M21 |
| --- | --- | --- | --- |
|  | b/se | b/se | b/se |
| Top 10% share | 1.032^**^ | -1.347 | -2.116^*^ |
|  | (0.324) | (1.157) | (0.920) |
| GDP per capita (log.) | -0.012 |  |  |
|  | (0.033) |  |  |
| GDP per capita |  | 0.010^+^ | 0.078 |
|  |  | (0.006) | (0.068) |
| Income tertile (ref. bottom) |  |  |  |
| Middle tertile | -1.334^***^ | -1.138^+^ | -1.057 |
|  | (0.030) | (0.614) | (1.333) |
| Top tertile | 0.281^***^ | -0.206 | -1.295 |
|  | (0.034) | (1.522) | (2.500) |
| SIOPS | -0.002^*^ | 0.000 | 0.000 |
|  | (0.001) | (0.001) | (0.001) |
| Education | 0.004 | -0.002 | -0.002 |
|  | (0.004) | (0.003) | (0.003) |
| Age | 0.002^*^ | 0.001 | 0.001 |
|  | (0.001) | (0.001) | (0.001) |
| Female (ref. male) | -0.005 | 0.011 | 0.011 |
|  | (0.025) | (0.027) | (0.027) |
| Middle tertile # Top 10% share |  | 1.576 | 3.289^**^ |
|  |  | (1.256) | (1.132) |
| Top tertile # Top 10% share |  | 4.743 | 7.472^***^ |
|  |  | (2.920) | (2.260) |
| Middle tertile # GDP per capita |  | -0.021^***^ |  |
|  |  | (0.006) |  |
| Top tertile # GDP per capita |  | -0.026 |  |
|  |  | (0.016) |  |
| Middle tertile # GDP per capita (log.) |  |  | -0.144 |
|  |  |  | (0.103) |
| Top tertile # GDP per capita (log.) |  |  | -0.084 |
|  |  |  | (0.189) |
| Constant |  | 1.060^+^ | 0.922 |
|  |  | (0.592) | (0.934) |
| *Random effects* |  |  |  |
| var(country) | 0.017^**^ | 0.164^**^ | 0.173^**^ |
|  | (0.006) | (0.056) | (0.060) |
| var(middle tertile) |  | 0.186^**^ | 0.232^**^ |
|  |  | (0.064) | (0.075) |
| var(top tertile) |  | 1.032^***^ | 1.121^***^ |
|  |  | (0.255) | (0.292) |
| cov(middle tertile, top tertile) | | 0.359^**^ | 0.425^**^ |
|  |  | (0.127) | (0.149) |
| cov(middle tertile, country) | | -0.162^**^ | -0.182^**^ |
|  |  | (0.060) | (0.067) |
| cov(top tertile,country) | | -0.376^**^ | -0.407^**^ |
|  |  | (0.116) | (0.127) |
| N_indiv_ | 31,368 | 31,368 | 31,368 |
| N_country_ | 29 | 29 | 29 |

*Notes: +P < 0.10; *P < 0.05; **P < 0.01; ***P < 0.001; weighted; cluster-robust standard errors in parentheses.*

*Source: Author's own calculations, ISSP 2019.*

Table OA5. Multilevel multinomial logistic regression of positioning bias using log. GDP and adding cross-level interactions with (log.) GDP

|  | M22 | | M23 | | M24 | |
| --- | --- | --- | --- | --- | --- | --- |
|  | Under | Over | Under | Over | Under | Over |
|  | b/se | b/se | b/se | b/se | b/se | b/se |
| Top 10% share | 4.004^***^ | -2.079^*^ | 5.432^**^ | -1.309 | 7.010^***^ | -1.963^*^ |
|  | (1.176) | (0.882) | (1.869) | (1.115) |  |  |
| GDP per capita (log.) | -0.091 | 0.049 |  |  | 0.138 | 0.083 |
|  | (0.090) | (0.055) |  |  | (0.182) | (0.063) |
| GDP per capita |  |  |  |  |  |  |
|  |  |  | -0.007 | 0.009 |  |  |
| Income tertile (ref. bottom) |  |  | (0.011) | (0.006) |  |  |
| Middle tertile | 2.297^***^ | -2.393^***^ | 4.194^***^ | -1.313^***^ | 6.843^***^ | -1.309 |
|  | (0.155) | (0.074) | (0.958) | (0.348) | (1.694) | (1.475) |
| Top tertile | 4.931^***^ | -5.044^***^ | 6.087^***^ | -2.827^*^ | 7.459^***^ | -2.995 |
|  | (0.168) | (0.305) | (1.098) | (1.213) | (1.873) | (2.985) |
| SIOPS | -0.008^**^ | 0.008^***^ | -0.008^**^ | 0.009^***^ | -0.009^**^ | 0.008^***^ |
|  | (0.003) | (0.002) | (0.003) | (0.002) | (0.003) | (0.002) |
| Education | -0.023^***^ | 0.015^***^ | -0.022^***^ | 0.016^***^ | -0.022^***^ | 0.015^***^ |
|  | (0.007) | (0.004) | (0.007) | (0.004) | (0.007) | (0.004) |
| Age | 0.001 | 0.002 | 0.001 | 0.002 | 0.001 | 0.002 |
|  | (0.002) | (0.002) | (0.002) | (0.002) | (0.002) | (0.002) |
| Female (ref. male) | -0.008 | 0.039 | -0.007 | 0.037 | -0.007 | 0.038 |
|  | (0.053) | (0.029) | (0.052) | (0.029) | (0.053) | (0.029) |
| Middle tertile # Top 10% share |  |  | -3.663^*^ | -1.716^*^ | -3.706^*^ | -0.498 |
|  |  |  | (1.772) | (0.753) | (1.630) | (0.928) |
| Top tertile # Top 10% share |  |  | -2.242 | 0.245 | -2.291 | 5.067^*^ |
|  |  |  | (2.053) | (2.458) | (1.758) | (2.499) |
| Middle tertile # GDP per capita |  |  | -0.014 | -0.013^***^ |  |  |
|  |  |  | (0.011) | (0.003) |  |  |
| Top tertile # GDP per capita |  |  | -0.007 | -0.054^***^ |  |  |
|  |  |  | (0.012) | (0.013) |  |  |
| Middle tertile # GDP per capita (log.) |  |  |  |  | -0.307^*^ | -0.089 |
|  |  |  |  |  | (0.127) | (0.121) |
| Top tertile # GDP per capita (log.) |  |  |  |  | -0.156 | -0.344 |
|  |  |  |  |  | (0.142) | (0.236) |
| Constant | -3.307^**^ | 0.601 | -4.599^***^ | 0.478 | -6.821^**^ | 0.215 |
|  | (1.283) | (0.764) | (0.966) | (0.560) | (2.289) | (0.860) |
| *Random effects* |  |  |  |  |  |  |
| var(over) |  | 0.306^***^ |  | 0.284^***^ |  | 0.315^***^ |
|  |  | (0.080) |  | (0.078) |  | (0.081) |
| var(under) |  | 0.160^***^ |  | 0.162^***^ |  | 0.161^***^ |
|  |  | (0.046) |  | (0.046) |  | (0.047) |
| cov(over, under) |  | -0.196^***^ |  | -0.196^***^ |  | -0.198^***^ |
|  |  | (0.056) |  | (0.055) |  | (0.058) |
| N_indiv_ | 31,368 |  | 31,368 |  | 31,368 |  |
| N_country_ | 29 |  | 29 |  | 29 |  |

*Notes: +P < 0.10; *P < 0.05; **P < 0.01; ***P < 0.001; weighted; cluster-robust standard errors in parentheses.*

*Source: Author's own calculations, ISSP 2019.*


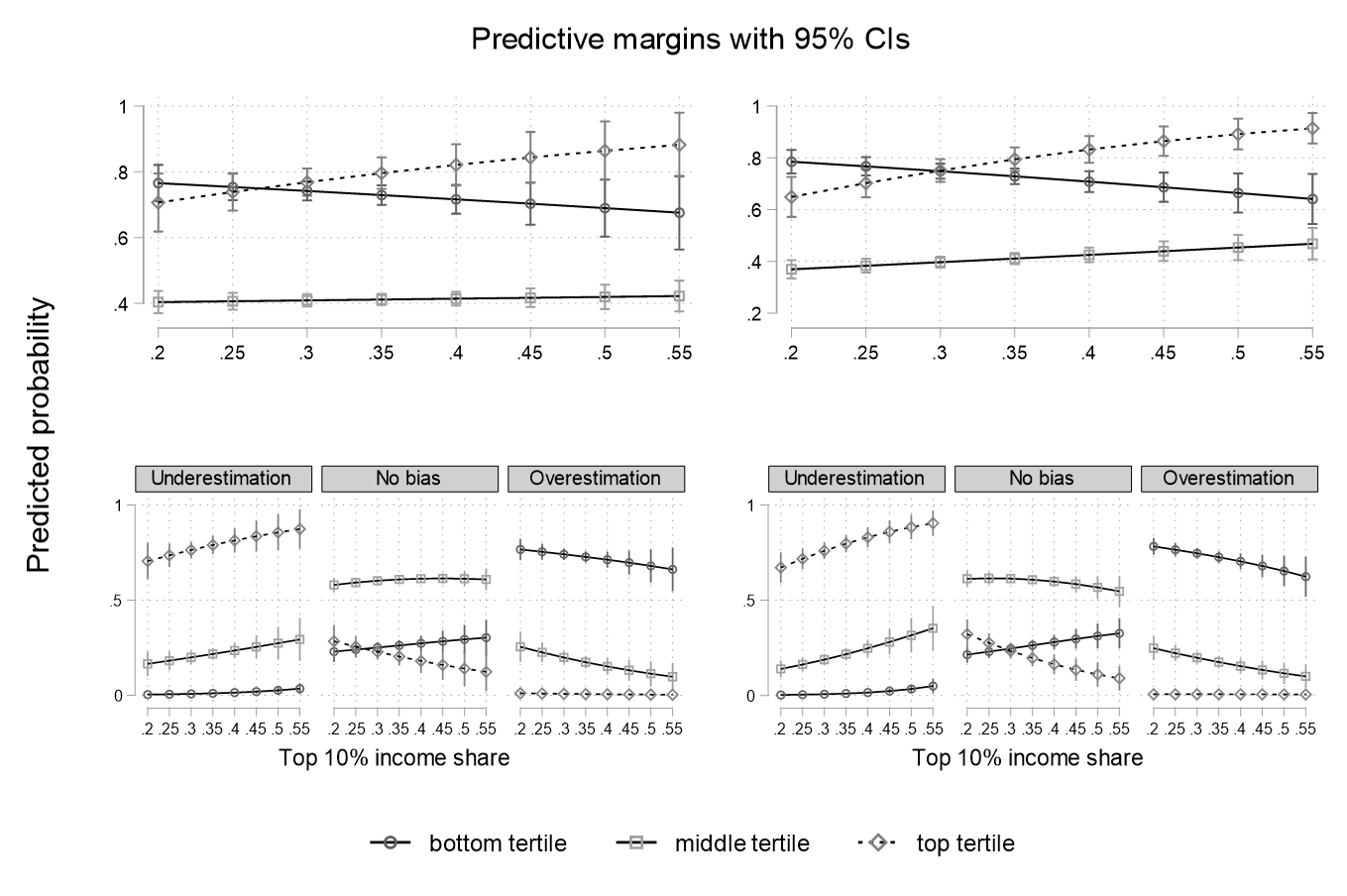


Figure OA2. Predicted probabilities of positioning bias over top 10% income share after controlling for (log.) GDP per capita x income tertiles (based on M20, M21, M23 and M24).


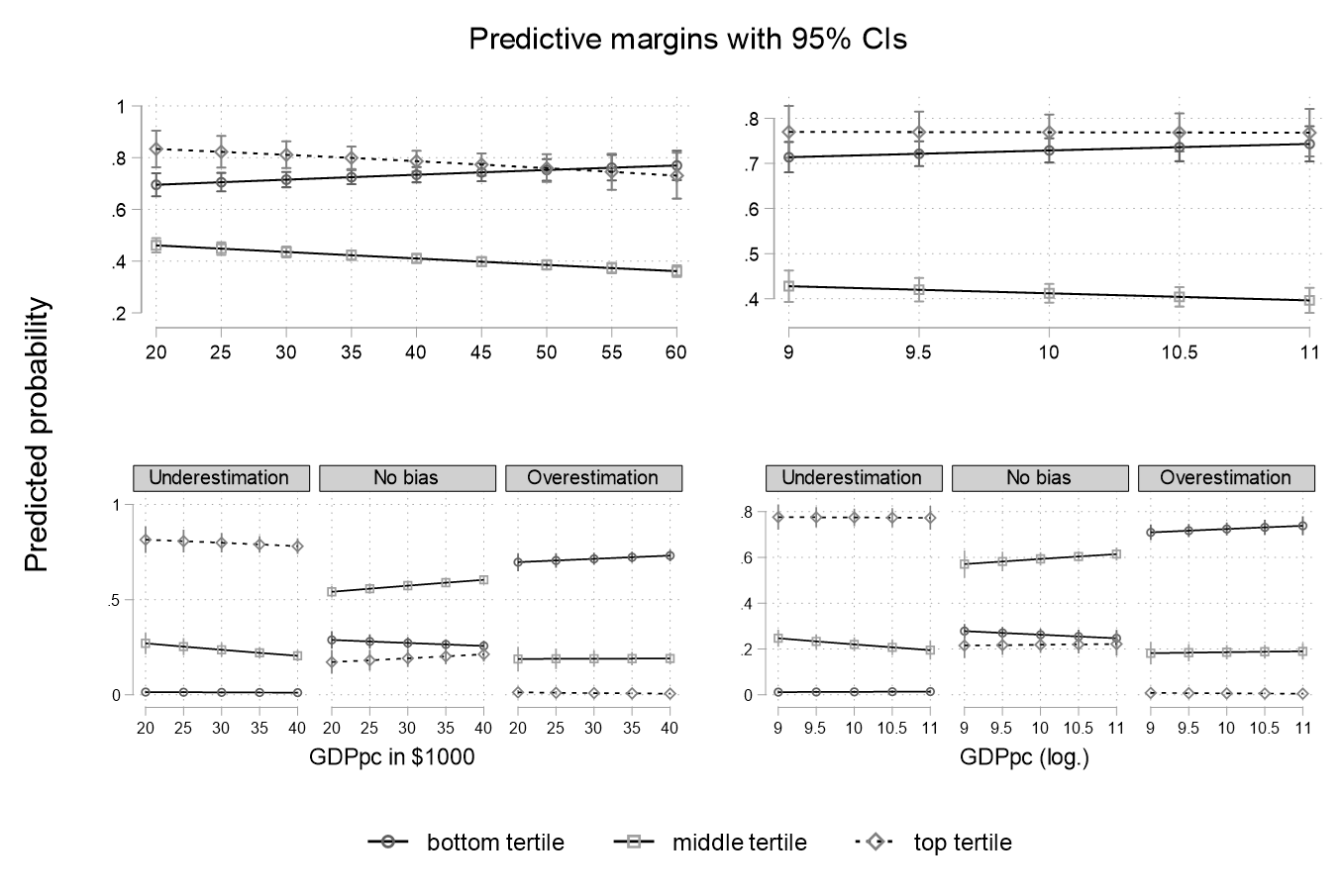


Figure OA3. Predicted probabilities of positioning bias over (log.) GDP per capita (based on M20, M21, M23 and M24).

Figure OA4. Average marginal effects of the top 10% income share.

*Notes: Base refers to the average marginal effect based on M3. Control displays the average marginal effect after adding the respective classification dummy to M3. Below that, the average marginal effects for the respective subsample are displayed, i.e., the effect of income inequality across early industrialized (AT AU CH DE DK FI FR GB IS NO SE US) or late industrialized (BG CL CZ HR IL IT JP LT NZ PH RU SI SR TH TW VE ZA) countries; developed (CL PH SR TH TW VE ZA) or developing (CL PH SR TH TW VE ZA) countries; Global North (AT AU CH DE DK FI FR GB IL IS IT JP LT NO NZ SE SI US), Global South (BG CL CZ HR PH RU SR TH TW VE ZA) countries; and above median GDP per capita (AT AU CH DE DK FI FR GB IL IS NO SE TW US) or below GDP per capita (BG CL CZ HR IT JP LT NZ PH RU SI SR TH VE ZA) countries.*

Table OA6. Multilevel logistic regression of positioning bias (M20 by above/below median GDP contexts)

|  | M25 | M26 |
| --- | --- | --- |
|  | GDPpc > median | GDPpc < median |
|  | b/se | b/se |
| Top 10% income share | -0.927 | -2.091** |
|  | (2.379) | (0.709) |
| GDP per capita | 0.002 | -0.001 |
|  | (0.017) | (0.007) |
| Income tertile (ref. bottom) |  |  |
| Middle tertile | -0.873 | -2.420*** |
|  | (1.077) | (0.575) |
| Top tertile | -0.905 | -1.829+ |
|  | (2.713) | (1.083) |
| SIOPS | -0.001 | 0.001 |
|  | (0.002) | (0.002) |
| Education | -0.003 | -0.000 |
|  | (0.004) | (0.004) |
| Age | 0.002 | 0.001 |
|  | (0.002) | (0.001) |
| Female (ref. male) | 0.011 | 0.007 |
|  | (0.042) | (0.035) |
| Middle tertile # Top 10% share | 0.245 | 3.691*** |
|  | (2.385) | (1.058) |
| Top tertile # Top 10% share | 3.222 | 7.254*** |
|  | (5.729) | (2.044) |
| Middle tertile # GDP per capita | -0.020 | -0.002 |
|  | (0.016) | (0.009) |
| Top tertile # GDP per capita | -0.008 | 0.002 |
|  | (0.036) | (0.018) |
| Constant | 1.479 | 1.530*** |
|  | (1.209) | (0.430) |
| *Random effects* |  |  |
| var(country) | 0.204* | 0.112* |
|  | (0.084) | (0.056) |
| var(middle tertile) | 1.233** | 0.662** |
|  | (0.385) | (0.239) |
| var(top tertile) | 0.234** | 0.055+ |
|  | (0.086) | (0.032) |
| cov(middle tertile, top tertile) | 0.419* | 0.209+ |
|  | (0.178) | (0.116) |
| cov(middle tertile, country) | -0.209* | -0.070+ |
|  | (0.085) | (0.042) |
| cov(top tertile, country) | -0.502** | -0.157+ |
|  | (0.174) | (0.083) |
| N_indiv_ | 15,158 | 16,210 |
| N_country_ | 14 | 15 |

*Notes: +P < 0.10; *P < 0.05; **P < 0.01; ***P < 0.001; weighted; cluster-robust standard errors in parentheses.*

*Source: Author's own calculations, ISSP 2019.*
